# Innovative Use of Bilateral Pedicle Advancement Flap for Post-auricular Defect Reconstruction

**DOI:** 10.7759/cureus.72840

**Published:** 2024-11-01

**Authors:** Jena C Jacobs, Mario J Sequeira

**Affiliations:** 1 Dermatology, A.T. Still University, Kirksville, USA; 2 Dermatology, Brevard Skin and Cancer Center, Rockledge, USA; 3 Dermatology, University of Miami Miller School of Medicine, Miami, USA

**Keywords:** advancement flap, basal cell carcinoma (bcc), bipedicle skin flap, dermatological surgery, mohs surgery, reconstructive flap surgery

## Abstract

In the field of reconstructive dermatological surgery, managing extensive defects in regions characterized by limited skin elasticity and exposed cartilage after Mohs surgery presents significant challenges. Unique situations arise where primary closure is impractical due to the size and/or location of the defect, necessitating alternative, complex reconstructive techniques. These techniques allow surgeons to preserve structural integrity and optimize aesthetic results. In this case, a 78-year-old white male presented with a nodular basal cell carcinoma on the right posterior auricle. He underwent Mohs surgery, which resulted in a 1.5 x 1.5 cm defect. The authors used a bilateral pedicle advancement flap, a technique not commonly used in dermatologic surgery, to close the resulting defect. This approach resulted in an excellent surgical outcome with no complications at the 10-month follow-up. This case highlights the bilateral pedicle advancement flap as a viable and effective reconstruction method for small to mid-sized posterior auricular defects.

## Introduction

Bipedicle advancement flaps, though infrequently discussed in dermatologic literature, are a valuable yet underutilized technique in traditional linear reconstruction. They are typically reserved for large defects primarily on large midline structures such as the scalp, forehead, nose, and chin to achieve excellent aesthetic outcomes while preserving the natural architecture of the region [1-3].

Mohs surgery is a common technique utilized for skin cancer removal. During this procedure, the surgeon carefully removes cancerous tissue layer by layer, examining each layer under a microscope during the procedure [4]. This allows for the precise removal of all cancerous cells while sparing as much healthy tissue as possible. Each tissue layer is examined to ensure the margins are clear of cancer cells. If cancer is found at the margins, the surgeon only removes tissue from the affected area, minimizing damage to healthy tissue. The post-surgical defects come in many shapes and sizes and can require complex repair mechanisms for closure, as seen in this case.

Due to the unique challenges posed by this region's limited skin elasticity and the presence of exposed cartilage, a more complex closure method was required. The authors used a bilateral pedicle advancement flap. Despite their rare use in facial and neck reconstructions, they are utilized in specific scenarios, such as bipedicle nasal vestibular skin advancement flaps, for lining full-thickness nasal defects [5]. Technically, they are designed adjacent to the defect and advanced at a right angle to the flap's linear axis, leaving a secondary defect often requiring a split-thickness skin graft [5]. While these flaps often require secondary defect management through skin grafting or secondary intention healing, they are advantageous for relocating defects and ensuring healthy tissue coverage over bone or hardware, making them a versatile and effective reconstructive option [6]. This technique involves parallel incisions perpendicular to the direction of advancement, creating a bridge of tissue that is advanced into the defect [7]. This utility comes in handy when repairing difficult areas, such as a post-auricular defect. These repairs are typically successful if tissue is grafted from a secondary site, by means of a transposition flap, or if they are left to close by secondary intention. Therefore, we aimed to use an alternative technique, recognizing that, to our knowledge, this approach had not been previously documented for use in this specific body region. We found that the bilateral pedicle advancement flap is a favorable reconstruction method for posterior auricular defects.

## Case presentation

**Figure 1:**
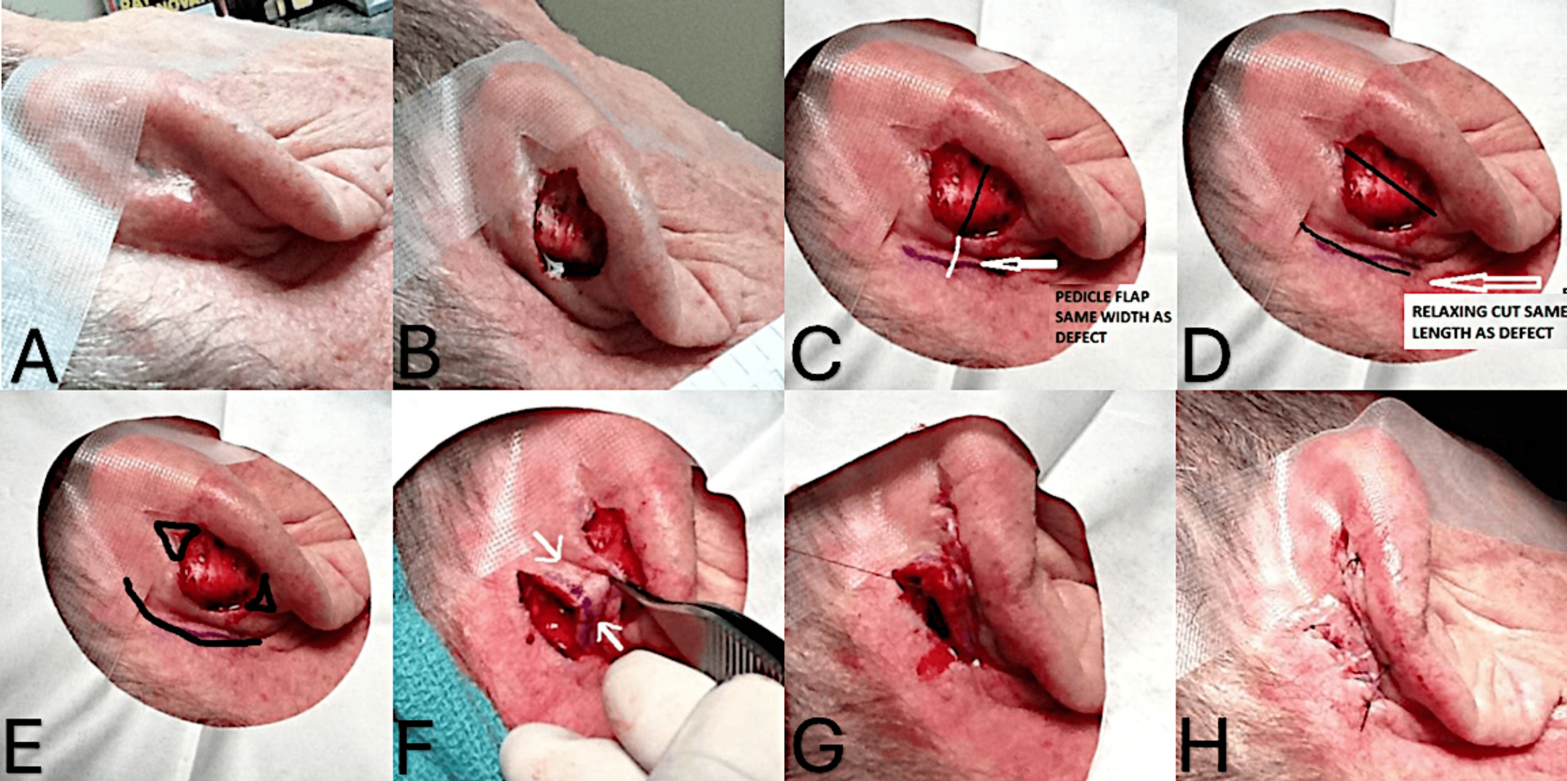
(a) Pearly plaque, 11 mm diameter on the right posterior auricle; (b) Mohs surgical defect (1.5 x 1.5 cm) after two stages to achieve clear margins; (c) measuring the width and length of the surgical defect; (d) the releasing incision was then made directly parallel and posterior a minimum of one width distance away from the defect; (e) small standing tissue cones superior and inferior to the defect; (f) undermining of the flap and secondary defect, arrows depict blood supply in both pedicles; (g) advancement of flap and closure of secondary defect; and (h) final closure

A 78-year-old white male was referred for an evaluation of a pearly patch, 11 mm diameter, on the right posterior auricle (Figure 1a). A shave biopsy was performed, and histopathological examination confirmed a basal cell carcinoma, nodular type. Mohs surgical excision was performed with a final circular defect measuring 1.5 x 1.5 cm after two stages to achieve clear margins (Figure 1b). The flap repair's initial step entailed measuring the surgical defect's width and length (Figure 1c). The releasing incision was then made directly parallel and posterior a minimum of one width distance away from the defect. This allowed the flap to be wide enough to cover the defect without undue lateral tension (Figure 1d) [6]. Small standing tissue cones superior and inferior to the defect need not be excised on the posterior auricular skin but may need to be in other body sites such as the superior forehead where they will be cosmetically more noticeable (Figure 1e) [7]. Undermining of the flap and secondary defect was made in the deep subcutaneous plane, and the flap was advanced onto the primary defect and repaired with an epidermal running 5-0 nylon suture (Figures 1f, 1g). The secondary defect was then closed with a layered repair using three subcutaneous 5-0 vicryl sutures and one running external 5-0 nylon suture (Figure 1h). Because the secondary defect may have a tendency to tent when repaired, a tacking subcutaneous suture may reduce the likelihood of this happening. Horizontal mattress sutures may also be required at the primary defect site repair to improve wound eversion.

**Figure 2:**
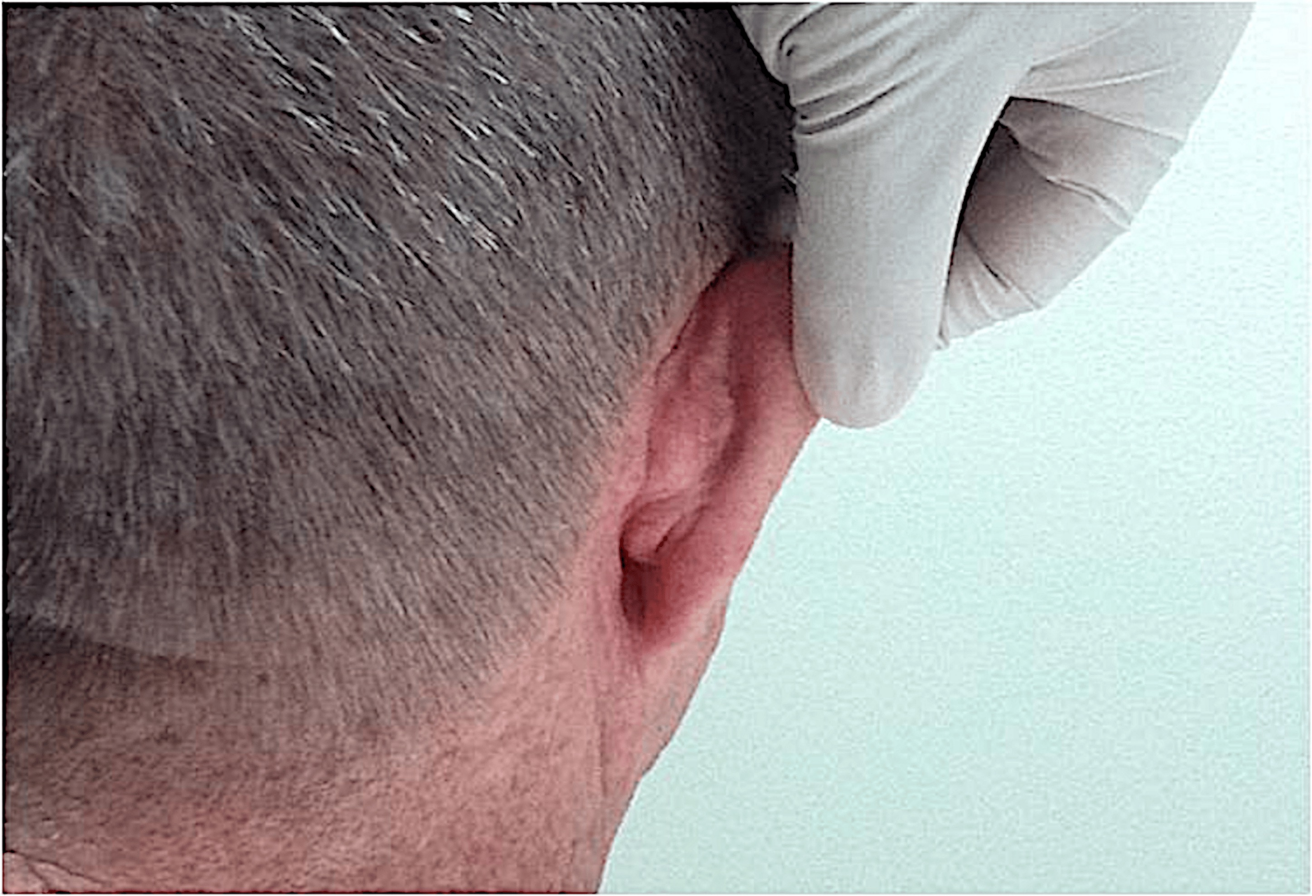
Two weeks post-procedure at suture removal

At the time of suture removal two weeks later (Figure 2) and 10-month follow-up, the surgical outcome was excellent. There were no post-operative complications such as flap necrosis or distortion of the auricle.

## Discussion

Surgical defects of the posterior auricle are generally challenging to repair using primary closure techniques due to the lack of skin elasticity in this region. The posterior auricle, being a relatively immobile area with tightly adherent skin overlying cartilage, does not provide the flexibility required for tension-free closure. Traditional repair methods commonly involve the use of full-thickness skin grafts or transposition flaps derived from the nearby posterior auricular sulcus, with the flaps being either superiorly or inferiorly based, depending on the defect’s location and size [5]. Full-thickness grafts are typically harvested from sites with sufficient donor skin, such as the supraclavicular or postauricular region, to ensure an adequate match in color and texture [5]. These techniques are effective but come with drawbacks, including donor site morbidity, prolonged healing time, and the risk of graft failure due to poor vascular supply in the recipient site [5].

In some situations, secondary intention healing may be considered, particularly for small or superficial defects [5]. This approach relies on the body’s natural healing processes to re-epithelialize the defect without surgical intervention. However, secondary intention is generally contraindicated when cartilage is exposed, particularly in cases where the perichondrium (the cartilage’s blood-supplying layer) is denuded [5]. In such instances, healing is significantly delayed, and the risk of complications like infection, or necrosis, increases. The absence of the protective perichondrium exacerbates these risks, as the exposed cartilage is particularly vulnerable to external insults, potentially leading to chronic inflammation or poor esthetic outcomes.

Given these challenges, the authors propose a more innovative approach utilizing a bilateral pedicle advancement flap. This technique offers an alternative that capitalizes on the inherent laxity of the auricular sulcus, a region that provides a moderate degree of mobility compared to the tightly adherent skin of the posterior auricle itself. The bilateral pedicle advancement flap involves making a single relaxing incision parallel and posterior to the defect [7]. This incision allows the advancement of skin onto the defect in a tension-free manner, effectively utilizing local tissue for coverage [7]. The bilateral nature of the flap ensures a robust blood supply from both sides of the pedicle promoting reliable healing and reducing the risk of necrosis [8].

This technique not only offers excellent vascular support to the flap, enhancing its viability, but also optimizes tissue utilization by minimizing the need for additional skin grafts or extensive flap mobilization [9]. By reducing the reliance on distant donor sites, this method significantly lowers donor site morbidity and accelerates the overall recovery process [9]. Moreover, the technique allows for the effective closure of secondary defects created by the relaxing incision, thus streamlining the overall surgical process and ensuring a more favorable cosmetic and functional outcome [10]. Ultimately, the bilateral pedicle advancement flap provides a versatile and effective solution for challenging posterior auricular defects, particularly in cases where traditional repair methods may not be ideal or where cartilage exposure precludes healing by secondary intention.

## Conclusions

In conclusion, the bilateral pedicle advancement flap presents a viable and effective option for reconstructing small to mid-sized defects of the posterior auricle, particularly when traditional methods are unsuitable due to the region's limited skin elasticity and the presence of exposed cartilage. The case demonstrated no post-operative complications and an outstanding surgical result at the 10-month follow-up, underscoring the flap's potential as a reliable reconstruction strategy in dermatologic surgery. By demonstrating the successful application of this flap in posterior auricle reconstruction, the authors contribute to expanding the repertoire of effective surgical techniques available to dermatologic and plastic surgeons. This approach not only challenges existing paradigms but also sets the stage for broader utilization of the bilateral pedicle advancement flap in clinical practice, ultimately promoting better esthetic and functional patient outcomes in auricular reconstruction.
